# Activation of stress-related signalling pathway in human cells upon SiO_2 _nanoparticles exposure as an early indicator of cytotoxicity

**DOI:** 10.1186/1477-3155-9-29

**Published:** 2011-07-29

**Authors:** Bashir Mustafa Mohamed, Navin Kumar Verma, Adriele Prina-Mello, Yvonne Williams, Anthony M Davies, Gabor Bakos, Laragh Tormey, Connla Edwards, John Hanrahan, Anna Salvati, Iseult Lynch, Kenneth Dawson, Dermot Kelleher, Yuri Volkov

**Affiliations:** 1 Department of clinical medicine, Institute of Molecular Medicine, Trinity College Dublin, Dublin8, Ireland; 2 Centre for Research on Adaptive Nanostructures and Nanodevices (CRANN), Naughton Institute, Trinity College Dublin, Dublin2, Ireland; 3 Glantreo Ltd., Environmental Research Institute (ERI) Building, Lee Road, Cork, Ireland; 4 Centre for BioNano Interactions, School of Chemistry and Chemical Biology, University College Dublin, Dublin4, Ireland

## Abstract

**Background:**

Nanomaterials such as SiO_2 _nanoparticles (SiO_2_NP) are finding increasing applications in the biomedical and biotechnological fields such as disease diagnostics, imaging, drug delivery, food, cosmetics and biosensors development. Thus, a mechanistic and systematic evaluation of the potential biological and toxic effects of SiO_2_NP becomes crucial in order to assess their complete safe applicability limits.

**Results:**

In this study, human monocytic leukemia cell line THP-1 and human alveolar epithelial cell line A549 were exposed to a range of amorphous SiO_2_NP of various sizes and concentrations (0.01, 0.1 and 0.5 mg/ml). Key biological indicators of cellular functions including cell population density, cellular morphology, membrane permeability, lysosomal mass/pH and activation of transcription factor-2 (ATF-2) were evaluated utilizing quantitative high content screening (HCS) approach and biochemical techniques. Despite the use of extremely high nanoparticle concentrations, our findings showed a low degree of cytotoxicity within the panel of SiO_2_NP investigated. However, at these concentrations, we observed the onset of stress-related cellular response induced by SiO_2_NP. Interestingly, cells exposed to alumina-coated SiO_2_NP showed low level, and in some cases complete absence, of stress response and this was consistent up to the highest dose of 0.5 mg/ml.

**Conclusions:**

The present study demonstrates and highlights the importance of subtle biological changes downstream of primary membrane and endocytosis-associated phenomena resulting from high dose SiO_2_NP exposure. Increased activation of transcription factors, such as ATF-2, was quantitatively assessed as a function of i) human cell line specific stress-response, ii) SiO_2_NP size and iii) concentration. Despite the low level of cytotoxicity detected for the amorphous SiO_2_NP investigated, these findings prompt an in-depth focus for future SiO_2_NP-cell/tissue investigations based on the combined analysis of more subtle signalling pathways associated with accumulation mechanisms, which is essential for establishing the bio-safety of existing and new nanomaterials.

## Background

Nanoparticles have received increasing attention for their potential applications in biology and medicine in recent years [1-3]. Notably, atmospheric particulates, such as diesel exhaust derivatives, have been recognized to have harmful effects on human health, including systemic and cardiovascular effects [4]. Lately, there has been a growing awareness of the need to elucidate the underlying interactions between cells and nanomaterials in parallel with the development of nanomaterials applications, in order to ensure the safe implementation of nanotechnologies. This has become increasingly emphasised by many research groups worldwide in a large number of publications, in recent years [2,3,5-18]. As silica nanoparticles (SiO_2_NP) are extensively used in the biomedical field, for instance as biosensors for simultaneous assay of glucose [1], biomarkers for leukaemia cell identification using optical microscopy imaging [17], drug delivery [19], DNA delivery [20,21], cancer therapy [22], and enzyme immobilization [23], it is important to understand any potential and unintended toxic, functional or signalling effects they may induce as a consequence of their increased cellular access, compared to their macroscale silica variants.

It has been reported that *in vivo*, in a mouse model, ultrafine colloidal silica particles (diameter < 100 nm) induce lung injury [24] and lung inflammation, which manifest as neutrophil accumulation at early stage of exposure (24 h) and chronic granulomatous inflammation at later stages (14 weeks) [25]. Furthermore, several studies have also provided evidence that SiO_2_NP cause abnormal clusters of topoisomerase I in the nucleoplasm of cells, and pro-inflammatory stimulation both *in vivo *and *in vitro *[26-29].

Lin et al. [30] demonstrated in an *in vitro *study that amorphous SiO_2_NP (15 and 46 nm) significantly reduced the viability of human alveolar epithelial cells A549 in a dose- and time- dependent manner. They also found that nanometre-sized SiO_2_NP inhibited DNA replication, transcription, and cell proliferation. Low toxicity induced by 200 nm-size (hereafter refer as nm only) SiO_2_NP was reported by Wottrich et al. [31]. Conversely, a study by Brunner et al. found that SiO_2_NP agglomerates (diameter > 200 nm) did not induce a toxic effect either *in vivo *or *in vitro *[32]. Yu et al. also reported that amorphous silica nanoparticles below 100 nm did not induce any cytotoxicity measured by the mitochondrial viability assay [33].

In nanomaterial toxicity the study of the interaction of the reporter assay dye compounds with nanoparticles may cause significant interference with the assay performance, for instance due to fluorescence shift [34]. Recently, a cell-based high content screening (HCS) assay operating on the principle of fully automated fluorescence microscopy was introduced as a modern drug discovery tool [35]. This technology is becoming an indispensable approach to research and industry, assisting in understanding complex cellular processes in disease pathogenesis [36], drug target validation and drug lead identification [37-39]. HCS assays are especially useful in studying cytotoxicity of nanomaterials, because they allow for multiplexing of key reporter parameters such as cell viability, permeability, membrane potential, and lysosomal mass/pH [17,40,41]. Therefore special considerations have been given for the experimental design of the cell-nanoparticles interaction assessment to standardise every operation and remove potential sources of inconsistency [5-7,40,42].

To elucidate whether the SiO_2_NP can induce stress-related damage in living cells, the activation of transcription factor-2 (ATF-2), following exposure to the SiO_2_NPs, was investigated. ATF-2 is a member of the basic region-leucine zipper transcription factor family that regulates the expression of genes in response to various stress signals, and it is known to acquire its transcriptional activity upon phosphorylation by MAP kinases, including JNK and p38 [43,44]. Because ATF-2 must be localised in the nucleus to induce gene expression, its translocation is a definitive measure of its activation, and marks an earlier event than reporter gene expression [44,45].

The present experimental study was designed to carry out a mechanistic and systematic multiparametric quantitative analysis of human cells responses to SiO_2_NP of various sizes and concentrations utilising automated HCS approach. Despite a low toxic response to SiO_2_NP by all cell types in this study, as assessed by cell growth, lysosomal mass/pH and cell membrane integrity, we registered activation of gene stress marker ATF-2 thereby indicating the triggering of stress-related signalling pathways prior to the onset of "classical" signs of cytotoxicity.

## Methods

### Reagents and Antibodies

Dulbecco's modified Eagle medium (DMEM), RPMI 1640 and foetal bovine serum were from Gibco (Invitrogen, BioSciences Ltd., Dublin, Ireland). HitKit™ for ATF-2 activation and multiparametric cytotoxicity assay 1 (MPCT1) were from Thermo Fisher Scientific (Thermo Fisher Scientific Inc., USA). Rabbit monoclonal anti-JNK, anti-phospho-JNK, anti-p38, anti-phospho- p38 and horseradish peroxidase conjugated anti-rabbit antibodies were from Cell Signaling Technology (Danvers, MA, USA). PVDF membrane was obtained from Pall Gelman Laboratories (Ann arbor, MI, USA). Acrylamide-bisacrylamide solution, Acrylogel (30%) was purchased from BDH (VWR International Ltd., UK). ECL plus reagent was purchased from Amersham (Arlington Heights, IL, USA). All other reagents were from Sigma (St Louis, MO, USA), unless indicated otherwise.

### Cell culture

Two human cell lines, one phagocytic and one non-phagocytic origin were used: a monocytic leukaemia THP-1 and an alveolar epithelial A549 (ATCC, Manassas, VA, USA). A549 cells were cultured in DMEM and THP-1 cells in RPMI 1640 medium. Both the culture media were supplemented with 10% foetal bovine serum, 200 mM L-glutamine, 10000 U/ml penicillin and 10 mg/ml streptomycin. For experimentation, A549 and THP-1 cells were seeded in 96-well plates at 5000 and 15000 respectively (Nunc, Inc., USA) and were maintained at 37°C and 5% CO_2_. THP-1 cells were stimulated with 25 ng/ml of phorbol 12-myristate 13-acetate for 72 h before SiO_2_NP exposure.

### Nanoparticles

Three amorphous SiO_2_NP of different sizes (30, 80, and 400 nm) (Glantreo Ltd., Cork, Ireland) were evaluated and compared to commercially available Sigma Ludox 40 nm, positively charged alumina coated chloride-ion stabilized SiO_2_NP and 20 nm, sodium counterion stabilised SiO_2_NP (Sigma-Aldrich, LUDOX CL 420883 and LUDOX CL 420891 respectively). The physico-chemical properties of all chosen nanoparticles such as size, surface charge and pH have been fully characterised and previously reported by Barnes et al. [46], (for reference see Table 1, Barnes et al.) as part of a multisite evaluation of nanoparticle complete characterization (Nanointeract project under the European Union Framework Programme 6). These SiO_2_NP were used to study the cellular toxic and stress responses in 6 and 96 well plates of adherent cells exposed the above listed SiO_2_NP at various concentrations (0.01, 0.1, and 0.5 mg/ml) for 1, 3, 6, and 24 h incubation. All assays were performed in triplicate. After exposure, the cells were washed three times with culture medium to remove any unbound and non-internalised nanoparticles. Qualitative imaging of the SiO_2_NP cell uptake was enabled by the use of fluorescently labelled SiO_2_NP (produced also by Glantreo Ltd.). These were synthesised via a co-condensation reaction where Rhodamine 6G soluble dye was incorporated into the silica framework during the synthesis of the nanoparticles. It is known that by the incorporation of the dye within the silica framework, the dye release is prevented by the lack of charge transfer which is usually associated with a surface functionalisation of the fluorescent dye [47]. Therefore, in our study when dispersed in biological, or water based solutions no obvious difference between the unlabelled and Rhodamine 6G labelled amorphous SiO_2_NP was found due to the complete amorphous nature of the mesoporous silica.

### High content screening and confocal microscopy

As mentioned, for the imaging of NP intracellular localisation two custom modified fluorescently labelled SiO_2_NP (30 nm and 400 nm) were used. To determine if SiO_2_NP are endocytosed by active or passive transport routes, cells were incubated at 37°C and 4°C, to monitor active and passive diffusion, respectively. THP-1 and A549 cells were incubated with 0.01, 0.1 and 0.5 mg/ml of these labelled SiO_2_NP for intervals ranging from 15 minutes to 24 h in a 37°C incubator with 5% CO_2_. Then, cells were washed in phosphate-buffered saline solution (PBS) at pH 7.4 and fixed in 3% paraformaldehyde (PFA). For the 4°C assay, cells were exposed to the 0.1 mg/ml of labelled SiO_2_NP (30 nm) for 24 h, and then cells were fixed with 3% PFA. In order to observe the impact of passive transport on SiO_2_NP uptake, cells were pre-treated with sodium azide for 3 h (0.1%, 0.015 M).

High resolution intracellular accumulation of fluorescently labelled nanoparticles was visualized by confocal laser scanning microscopy (Carl Zeiss, Axiovert, Germany). Two channel qualitative imaging was carried out by acquiring a series of Z-stack images to verify the accumulation of the particles within the cells as a function of particle concentration and exposure time. Cellular uptake of labelled SiO_2_NP (time-course and dose-range) was further imaged and quantified using an automated IN Cell Analyzer 1000 HCA platform; (GE Healthcare, UK) and IN Cell Investigator software (GE Healthcare, UK), respectively.

### Multiparameter cytotoxicity assay using HCS

A multiparametric cytotoxicity assay was performed using Cellomics^® ^HCS reagent HitKit™ as per manufacturer's instructions (Thermo Fisher Scientific Inc., USA). This kit measures cell viability, cell membrane permeability and lysosomal pH which are toxicity-attributed phenomena. Variations in cell membrane permeability, measured as changes in luminescence intensity, indicated an enhancement of cell membrane damage and decreased cell viability. It is known some toxins can interfere with the cell's functionality by affecting the pH of organelles such as lysosomes and endosomes, or by causing an increase in the number of lysosomes. The dye used in the chosen cytotoxicity assay is a weak base that accumulates in acidic organelles, such as lysosomes and endosomes, which allows changes in lysosomal physiology to be determined. For instance, an increase or decrease in ph of acidic organelles and the changes in lysosome numbers by compound toxicity results in a decrease or an increased of fluorescence intensity, respectively.

In agreement with a previous study, we took a toxicity reference set by treating the cells with cisplatin (10 nM, Sigma-Aldrich), which is a platinum-based chemotherapy drug used to treat various types of cancers, including sarcomas, some carcinomas (e.g. small cell lung cancer, and ovarian cancer), lymphomas, and germ cell tumours [48]. The experimental layout for the automated microscopic analysis, based on the In Cell analyzer 1000, was composed of untreated, cisplatin treated, and SiO_2_NP treated plates. All these were scanned and acquired in a stereology configuration of 6 randomly selected fields. Images were acquired at 10 X magnification using three detection channels with different excitation filters. These included a DAPI filter (channel 1), which detected blue fluorescence indicating nuclear intensity at a wavelength of 461 nm; FITC filter (channel 2), which detected green fluorescence indicating cell permeability at a wavelength of 509 nm and a TRITC filter (channel 3), which detected lysosomal mass and pH changes with red fluorescence at a wavelength of 599 nm.

The rate of cell viability and proliferation were assessed by the automated analysis of the nuclear count and morphology (DAPI filter); in parallel to the fluorescent staining intensities reflecting cell permeability (FITC filter) and lysosomal mass/pH changes (TRITC filter) were also quantified for each individual cell present in the examined microscopic fields by IN Cell Investigator (GE Healthcare, UK).

### ATF-2 Activation Assay using HCS

ATF-2 activation was measured using Cellomics HitKit^® ^as per manufacturer's instructions (Thermo Fisher Scientific Inc., USA). Briefly, cells seeded in 96-wells plates as described above were incubated with the above mentioned SiO_2_NP, for different intervals as previously indicated in the text, and in addition anisomycin was used as positive control (as supplied within the HitKit). For MAPK inhibition assay cells were pre-treated for 30 min with specific inhibitors for p38 (pyridinyl imidazole SB202190) or JNK (anthrapyraxolone SP600125) (Calbiochem, La Jolla, CA, USA). Exposed cells were then, washed in PBS, fixed with 3% PFA and stained for ATF-2 and nuclei (Hoechst). Plates were scanned, as previously described by using the principle of stereology in a randomly selected number of fields, using automated microscope (IN Cell Analyzer 1000 HCS platform, GE Healthcare, Buckinghamshire, UK) and images were acquired at 10 X magnification. Nuclear translocation of ATF-2 was quantified by IN Cell Investigator software using ad hoc nuclear trafficking analysis module (GE Healthcare, UK).

### Cell Lysis and Immunoblotting

Exposed cells were washed with ice-cold PBS and lysed at 4°C for 30 min in 50 mM HEPES buffer (pH 7.4) containing NaCl 150 mM, MgCl_2 _1.5 mM, EGTA 1 mM, sodium pyrophosphate 10 mM, sodium fluoride 50 mM, β-glycerophosphate 50 mM, Na_3_VO_4 _1 mM, 1% Triton X-100, phenylmethylsulphonyl fluoride 2 mM, leupeptin 10 μg/ml and aprotinin 10 μg/ml. The resulting lysates were centrifuged at 16,000 × *g *for 15 min at 4°C and the protein content of the supernatants was determined by the Bradford assay. Cell lysates were boiled in Laemmli buffer (final concentration: Tris-HCl 62.5 mM, pH 6.7, Glycerol 10% v/v, sodium dodecyl sulphate 2% w/v, bromophenol blue 0.002% w/v and 143 mM β-mercaptoethanol) for 5 min. Equal amounts of lysates were resolved by sodium dodecyl sulphate polyacrylamide gel electrophoresis (SDS-PAGE). The separated proteins were electrophoretically transferred to polyvinylidene fluoride (PVDF) membrane by semi-dry blotting for 1 h. The PVDF membranes were blocked in 5% non-fat dry milk in PBS Tween20 (PBST) [0.1% (v/v) Tween20 in phosphate buffered saline (PBS)] for 1 h at room temperature. After washing, the blots were incubated with the indicated primary antibodies (diluted according to manufacturer's instructions) overnight at 4°C with gentle rocking. After three washes in PBST, the membranes were incubated with the horseradish peroxidase conjugated secondary antibodies for 1 h at room temperature. The immunoreactive bands were visualized using an enhanced chemiluminescence detection system (Amersham, Arlington Heights, IL, US) and subsequent exposure to Kodak light sensitive film (Cedex, France).

### Statistical analysis

The response of the two cell lines to the chosen SiO_2_NP sizes and concentrations was analyzed by 2-way ANOVA with Bonferroni post-test analysis with GraphPad Prism v4 (GraphPad Software, USA). A *p*-value of <0.05 was considered to be statistically significant. For the multiparametric analysis, due to the large amount of information acquired a data mining and exploration platform was used (KNIME (http://KNIME.org, 2.0.3) in combination with a screening module HiTS (http://code.google.com/p/hits, 0.3.0) in order to screen and normalised all parameters under investigation, as previously reported [49,50]. All measured parameters were normalised using their respective percent of the positive controls. Z score was used for scoring the normalised values. These scores were summarised using the mean function as follows Z score = (x-mean)/StDev, as from previous work [51]. Heatmaps graphical illustration in a colorimetric gradient table format was adopted as most suitable schematic representation to report on any statistical significance and variation from normalised controls based on their Z score value. Heatmaps tables illustrate the range of variation of each quantified parameter from the minimum (green) through the mean (yellow) to the maximum (red) accordingly to the parameter under analysis.

### Densitometric Analysis

Densitometric analyses of the western blots were performed as described previously [52] using GeneTools software (Syngene, Cambridge, UK). The relative values of the samples were determined by normalising all data to the respective untreated control samples of each experiment.

## Results

### Cellular uptake of SiO_2_NP

**Figure 1:**
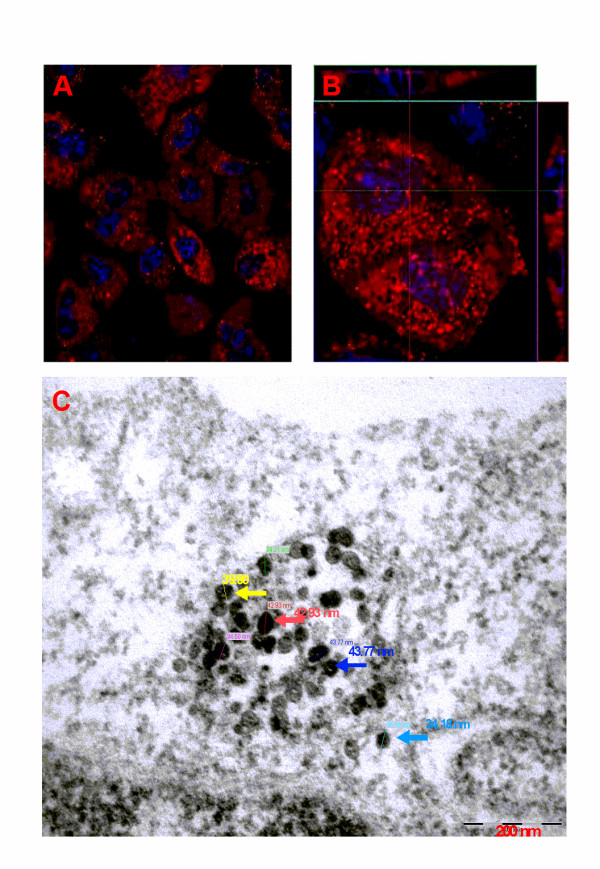
**Confocal microscopic image of A549 cells showing SiO_2_NP uptake**. (A) A549 cells growing on Permanox^® ^chamber slide were incubated with Rhodamine labelled SiO_2_NP (30 nm) for 24 h. After this time, culture media was carefully removed, cells were washed in PBS and cells were fixed in 3% paraformaldehyde. Nuclei were stained with Hoechst (blue). A representative sample population of cells were visualized by confocal microscopy using a 63 X oil immersion lens. (B) Three dimensional image stacks showing cytosolic accumulation of 30 nm SiO_2_NP (Z-stack = 27 slices at 0.45 μm per slices, Z-height 12.15 μm), (top image = x-z plane; centre image = x-y plane; right image = y-z plane). (C) Transmission Electron Microscope (TEM) image shows SiO_2_NP cytoplasmic accumulation, particle size distribution within an A549 epithelial lung cell (magnification 10 x and 120 KeV accelerating voltage).

Two cell lines, a human monocytic leukemia cell line THP-1 and a human alveolar epithelial cell line A549 were chosen to represent a physiologically relevant model of the *in vivo *first line of interaction between nanoparticles and human tissues, as it would be expected following exposure of the lungs (inhalation) and uptake of foreign material by phagocytic system utilizing innate immunity mechanisms. Confirmation of fluorescently labelled SiO_2_NP (30 nm and 400 nm) uptake by both THP-1 and A549 cell lines was observed by confocal microscopy after 24 h (Figure 1A, 1B). Transmission electron microscopy (TEM) imaging clearly show the absence of significant aggregated clusters of 40 nm size alumina coated particles (Figure 1C) where particle size measurements have been also included for clarity. Further to this, Light Scattering measurement was also carried out on retrieved 40 nm particles, and this did not show any significant aggregation as shown in (Additional file 1, Figure S1).

**Figure 2:**
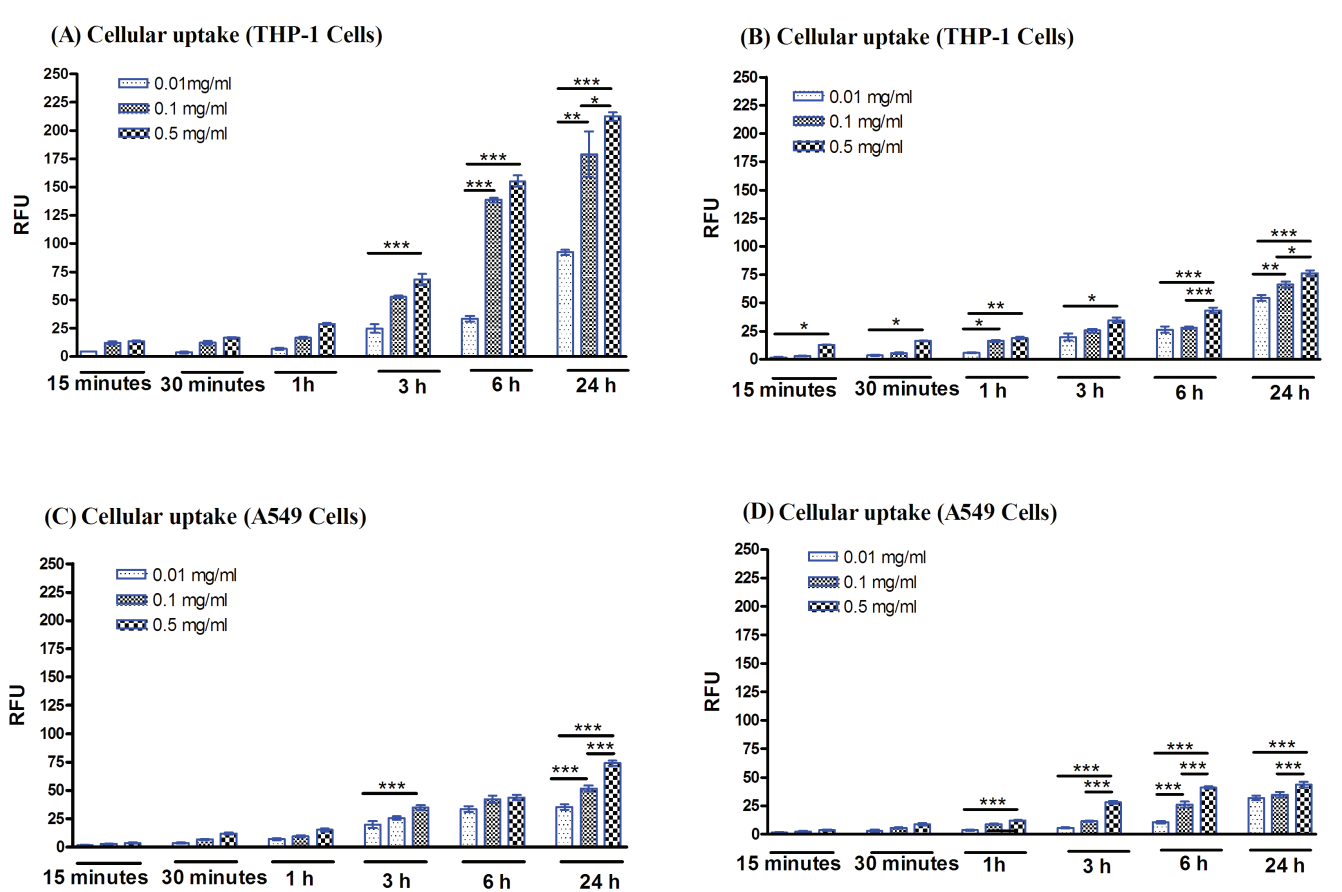
**Cellular uptake of 30 nm and 400 nm SiO_2_NP**. THP-1 (A, B) and A549 (C, D) cells growing on 96-well plates were exposed to various concentrations (0.01, 0.1, or 0.5 mg/ml) of 30 nm (A, C) or 400 nm (B, D) SiO_2_NP for multiple time points ranging from 15 min to 24 h. High content screenings analysis for the cytosolic accumulation of these particles was performed using an automated IN Cell Analyzer 1000 microscope and IN Cell Investigator image analysis software. Relative fluorescence intensity (RFU) represents the average intensity value of cytosolic accumulation of these labelled nanoparticles when measured in PBS at pH 7.4. Data shown is normalised to untreated control and presented as mean values of three independent experiments performed in triplicate samples. 2-way ANOVA with Bonferroni post-test analysis was carried out on the experimental data, normalised to controls, and statistically significant data is reported by "*" symbol, for *p  *< 0.05; "**" *p  *< 0.01; "***" for *p  *< 0.001.

**Figure 3:**
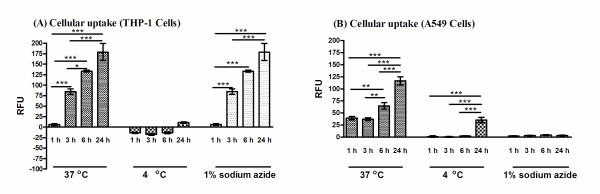
**Cellular uptake under low temperature condition and in the presence of metabolic inhibitor**. THP-1 (A) and A 549 (B) cells were exposed to 0.01 mg/ml of 30 nm SiO_2_NP over 1- 24 h time pried at 37°C, 4°C and 0.1% sodium azide. High content screening analysis for the cytosolic uptake of these particles was performed using an automated IN Cell Analyzer 1000 microscope and IN Cell Investigator image analysis software. Relative fluorescence intensity (RFU) represents the average of intensity value of cytosolic accumulation of these labelled nanoparticles when measured in PBS at pH 7.4. Data shown is normalised to untreated control and presented as mean values of three independent experiments performed in triplicate samples. Statistical analysis was carried out by 2-way ANOVA with Bonferroni post-test analysis and statistically significant data is reported by "*" symbol, for *p  *< 0.05; "**" *p  *< 0.01; "***" for *p  *< 0.001.

The evaluation of the rate of the cellular uptake of the labelled SiO_2_NP, versus untreated controls, relative fluorescent intensity was quantified after 24 h exposure (Figure 2, 3). As expected for all SiO_2_NP, THP-1 cells rapidly engulfed these nanoparticles, with a maximum uptake for 30 nm labelled SiO_2_NP at 0.1 mg/ml and higher concentrations (0.5 mg/ml). When comparing the accumulation rate, THP-1 cells engulfed the SiO_2_NP at a faster rate than the A549 cells which is totally acceptable due to the specialist phagocytic nature of the THP-1 cells. In addition for both cell lines, the intracellular accumulation of the 400 nm-size SiO_2_NP was slower when compared to the 30 nm particles. Next, we investigated whether the cellular uptake of the SiO_2_NP was mediated by an energy-dependent mechanism; thus the cells were incubated at both 37°C and 4°C up to 24 h with fluorescently labelled 30 nm SiO_2_NPs (0.1 mg/ml). THP-1 and A549 cells incubated at 4°C exhibited a significant reduction in the SiO_2_NP uptake compared to the equivalent incubation at 37°C (Figure 3A, 3B). Moreover, by blocking the active transport mechanism of the A549 cells by sodium azide treatment this significantly impeded SiO_2_NP uptake, as shown in Figure 3B; this was not the case for the THP-1 cell line where the sodium azide was not sufficiently adequate to block the SiO_2_NP uptake (Figure 3A). However, increased concentrations of sodium azide significantly blocked the uptake of SiO_2_NP (data not shown).

### Cell viability and proliferation assessment in response to SiO_2_NP

**Figure 4:**
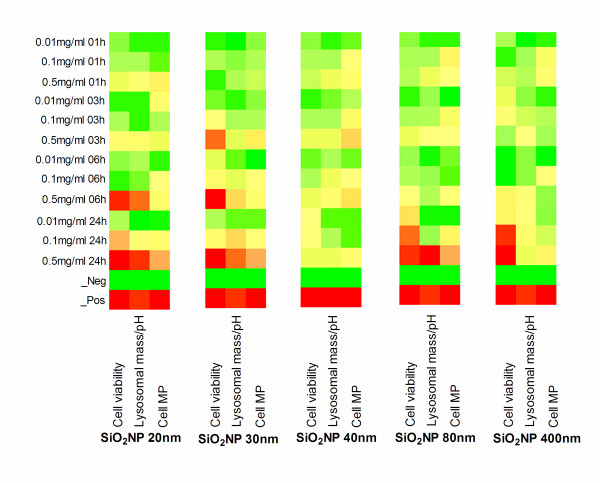
**Heatmaps tables illustrating toxicity indicated parameters in THP-1 cells exposed to SiO_2_NP**. THP-1 cells growing on 96-well plates were exposed to various concentrations (0.01, 0.1, or 0.5 mg/ml) of 20 nm (A), 30 nm (B), 40 nm (C), 80 nm (D), or 400 nm (E) SiO_2_NP for 1 h, 3 h, 6 h, or 24 h. Multiparametric high content screening analysis for cell count (left column), Lysosomal mass/pH (middle column) and cell membrane permeability (MP) (right column) was performed using an automated IN Cell Analyzer 1000 microscope and IN Cell Investigator image analysis software. Data represents three independent experiments performed in triplicate samples. Heatmaps were generated for the above indicated parameters and their colorimetric gradient table spans from: Dark green = lower than 15% of maximum value measured; Bright green = 30%; Yellow = 50%; Bright Orange = 60%; Dark Orange = 75%; Red = higher than 75% of maximum value. Cell viability colour gradients read as percentage of cell loss compared to normalised control (green = low cell viability loss, red = high loss) compared to normalised control. Heatmaps values are normalised using the percent of the positive controls and, Z score was calculated as described in the statistical analysis section.

**Figure 5:**
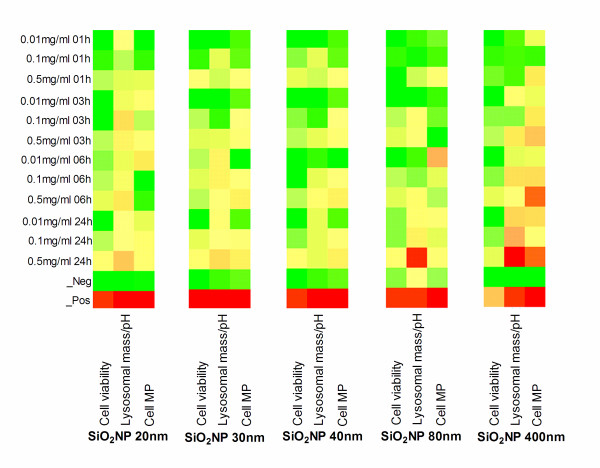
**Heatmaps tables illustrating toxicity indicated parameters in A549 cells exposed to SiO_2_NP**. A549 cells growing on 96-well plates were exposed to various concentrations (0.01, 0.1, or 0.5 mg/ml) of 20 nm (A), 30 nm (B), 40 nm (C), 80 nm (D), or 400 nm (E) SiO_2_NP for 1 h, 3 h, 6 h, or 24 h. Multi-parameter high content screening analysis for cell count, Lysosomal mass/pH and cell membrane permeability (MP) was performed using an automated IN Cell Analyzer 1000 microscope and IN Cell Investigator image analysis software. Data represents three independent experiments performed in triplicate samples. Heatmaps colorimetric gradient table for the A549 results span from: Dark green = lower than 15% of maximum value measured; Bright green = 30%; Yellow = 50%; Bright Orange = 60%; Dark Orange = 75%; Red = higher than 75% of maximum value. Cell viability colour gradients read as percentage of cell loss compared to normalised control (green = low cell viability loss, red = high loss) compared to normalised control. Heatmaps values are normalised using the percent of the positive controls and, Z score was calculated as described in the statistical analysis section.

The assessment of cell-SiO_2_NP interaction by means of viability and proliferation of THP-1 and A549 cells respectively was performed by HCS on the all the chosen SiO_2_NP (20, 30, 40, 80, 400 nm) as fully described in the material section. In addition, for each particle size the results are presented in colorimetric gradients (heatmap format table), as shown in Figure 4 and 5 for THP-1 and A549 respectively and also by statistical analysis (Additional file 1, Table S1a-c). THP-1 did not show any obvious viability reduction up to 6 h when compared to the untreated cellular control (Figure 4, "cell viability" column for each particle size analysed (A, B, C, D, E, incubation time increasing from top to bottom). There, a significant decrease in the cell viability was seen for the sodium counterion stabilised (20 nm) SiO_2_NP at 0.1, or 0.5 mg/ml (Figure 4A), for the 30 nm SiO_2_NP at 0.5 mg/ml (Figure 4B), and for the 80 nm or 400 nm SiO_2_NP at 0.01, 0.1, or 0.5 mg/ml (Figure 4D, 4E). Interestingly, no significant effect in the THP-1 cell viability was recorded for any of the tested doses of the alumina coated positively charged SiO_2_NP (40 nm) (Figure 4C) up to 24 h, also confirmed by their statistical analysis tables (Additional file 1, Table S1a-c). Conversely to the THP-1 cell line, no detectable effect on A549 cell viability was observed by any of the SiO_2_NP type or doses investigated in this study (Figure 5A-E).

### Changes in cell membrane permeability in response to SiO_2_NP

Further assessment of cell-SiO_2_NP interaction by means of cellular membrane permeability tests on THP-1 and A549 cells was performed by HCS on the all investigated SiO_2_NP (20, 30, 40, 80, 400 nm). In fact, it is known that the alterations of the cellular membrane permeability indicate the alterations of the physical condition of the cells [53]. For THP-1 cells the cellular membrane permeability was significantly increased over 24 h exposures to 20, 30 or 80 nm-size SiO_2_NP at 0.5 mg/ml concentration (Figure 4A, 4B, 4D). On the other hand, no significant changes were seen for the 40 nm and 400 nm SiO_2_NP at any concentrations (Figure 4C, 4E).

For the A549 cells at concentration of 0.5 mg/ml, no significant changes in the cell membrane permeability was seen upon exposure to 20, 30, 40 and 80 nm SiO_2_NP at all tested doses and incubation times when compared to the untreated control cells (Figure 5A-D) and confirmed by ANOVA statistical test (Additional file 1, Table S2a-c). In contrast, the 400 nm-size SiO_2_NP caused cell membrane alteration at different incubation times, as shown in Figure 5E

### Changes in lysosomal mass/pH in response to SiO_2_NP

The assessment of cell lysosomal mass/pH in response to a range of SiO_2_NP with different sizes (20, 30, 40, 80, 400 nm) was performed by HCS tool. A decrease or an increase of lysosomal mass/pH can designate an increased rate of the cytotoxicity. In this study, no significant changes were detected in lysosomal mass/pH staining intensity up to 6 h exposure to 20, 30, 80 nm SiO_2_NP at any of the concentrations tested in THP-1 cells. Conversely, the lysosomal mass/pH was markedly diminished in the THP-1 cells at the highest concentration (0.5 mg/ml) of the (20, 30 or 80 nm nanoparticles over 24 h exposure time (Figure 4A, 4B, 4D). However, 40 nm alumina coated SiO_2_NP and 400 nm-size uncoated SiO_2_NP did not induce any lysosomal mass/pH staining intensity changes for all tested doses in the THP-1 cell line, (Figure 4C, 4E). The lysosomal mass/pH staining intensity was increased in A549 cells following 24 h exposure to 20 and 80 nm SiO_2_NP at 0.5 mg/ml (Figure 5A, and 5D) and to 400 nm-size SiO_2_NP at 0.1 and 0.5 mg/ml concentrations (Figure 5E). Conversely, 40 nm alumina-coated SiO_2_NP did not induce any changes in the lysosomal mass/ph at any investigated concentrations in the A549 cells (Additional file 1, Table S3a-c).

### SiO_2_NP induces ATF-2 nuclear translocation in cultured cells

**Figure 6:**
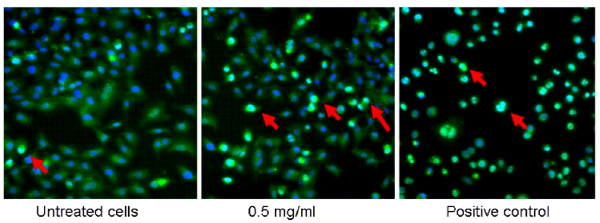
**Effect of SiO_2_NP on ATF-2 translocation in A549 cells**. A549 cells were exposed to 0.5 mg/ml SiO_2_NP (30 nm) or anisomycin (positive control) for 24 h and fixed in 3% paraformaldehyde. Cells were labelled with the Cellomics^® ^HCS reagent kit for ATF-2 activation (green). Nuclei were stained with Hoechst (blue). Cellular images were acquired by an IN Cell Analyzer 1000 automated microscope using 10 X objective (Image size: 0.897 mm × 0.671 mm). Red arrows indicate representative cells with activated ATF-2.

**Figure 7:**
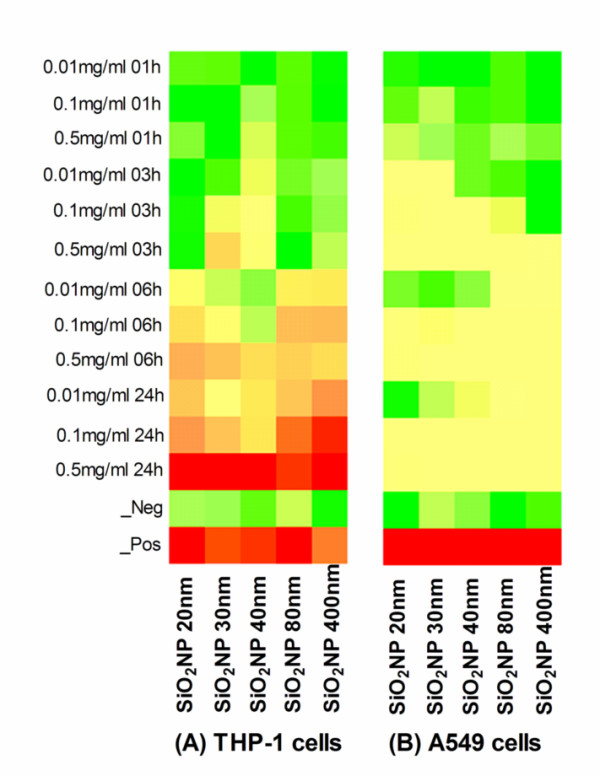
**Heatmaps tables illustrating SiO_2_NP induced nuclear translocation of ATF-2**. THP-1 (A), or A549 (B) cells growing on 96-well plates were exposed to various concentrations (0.01, 0.1, or 0.5 mg/ml) of 20 nm, 30 nm, 40 nm, 80 nm, or 400 nm SiO_2_NP for 1 h, 3 h, 6 h, or 24 h. Cells were labelled with the Cellomics^® ^HCS reagent kit for ATF-2 activation. High content screening analysis for nuclear translocation of ATF-2 was performed using an automated IN Cell Analyzer 1000 microscope equipped with IN Cell Investigator image analysis software that quantifies nuclear to cytoplasmic fluorescence intensity. Data represents three independent experiments performed in triplicate samples. Heatmaps colorimetric gradient table for the A549 results span from: Dark green = lower than 15% of maximum value measured; Bright green = 30%; Yellow = 50%; Bright Orange = 60%; Dark Orange = 75%; Red = higher than 75% of maximum value. Heatmaps values are normalised using the percent of the positive controls and, Z score was calculated as described in the statistical analysis section.

**Figure 8:**
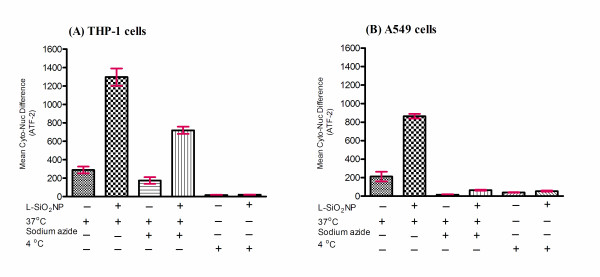
**Effect of labelled 30 nm SiO_2_NP on ATF- translocation at 37°C, under low temperature condition and in the presence of metabolic inhibitor**. THP-1 (A), or A549 (B) cells were growing on 96-well plates and exposed to labelled 30 nm SiO_2_NP (L-SiO_2_NP) for over 24 h pried at 37°C, 4°C and 0.1% sodium azide. Cells were labelled with the Cellomics^® ^HCS reagent kit for ATF-2 activation. High content screening analysis for nuclear translocation of ATF-2 was performed using an automated IN Cell Analyzer 1000 microscope equipped with IN Cell Investigator image analysis software that quantifies nuclear to cytoplasmic fluorescence intensity. Statistical analysis was carried out by 2-way ANOVA with Bonferroni post-test analysis and statistically significant data is reported by "*" symbol, for *p  *< 0.05; "**" *p  *< 0.01; "***" for *p  *< 0.001.

To assess whether cells exposed to any of the SiO_2_NP under investigation showed gene stress response, we measured ATF-2 activation by nuclear translocation. A549 and THP-1 cells were exposed to SiO_2_NP for various time points (1, 3, 6, or 24 h) and ATF-2 nuclear translocation was measured by HCS system. ATF-2 was absent in the nuclei of untreated A549 cell (Figure 6) and THP-1 cells (data not shown). Quantitative analysis by HCS demonstrated that in both cell types ATF-2 underwent nuclear translocation upon nanoparticles exposure. The nuclear translocation of ATF-2 was size-dependent across the SiO_2_NP tested. For A549 cells, it resulted in a clear incremental dose-translocation proportion, starting from 3 h exposure; whereas for THP-1 cells this phenomenon was obvious after 6 h. In both cases it reached a plateau at 24 h exposure (Figure 7). Despite the similar dynamics of ATF-2 activation registered for both the cell lines, the overall activation level was lower in A549 cells than that observed in THP-1 cells (Figure 7B vs 7A). In addition, labelled SiO_2_NP also induced ATF-2 activation in both THP-1 and A549 cells (Figure 8A, 8B). This was not observed following the blocking of active transport mechanisms in THP-1 and A549 cell lines either by sodium azide treatment or by low temperature conditions (4°C)

### SiO_2_NP induced activation of ATF-2 is dependent on JNK and p38

**Figure 9:**
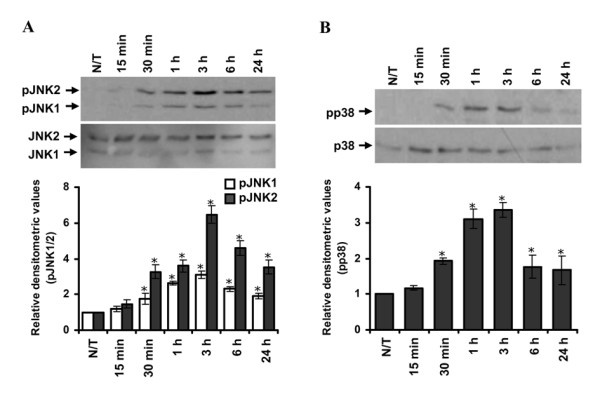
**Effect of SiO_2_NP on the phosphorylation of JNK1/2 and p38 in human lung epithelial cells**. A549 cells were treated with 80 nm SiO_2_NP for multiple time points ranging from 15 min to 24 h or left untreated (N/T) and lysed. Cell lysates (20 μg each) were resolved by SDS-PAGE and after Western blotting probed with anti-phospho-JNK1/2 or JNK1/2 (A), or anti-phospho-p38 or p38 (B). Relative densitometric analysis of the individual bands was performed and presented. Data are mean ± S.E.M. of three independent experiments. 2-way ANOVA with Bonferroni post-test analysis was carried out on the experimental data, with respect to corresponding controls, and statistically significant data is reported by "*" symbol, for *p  *< 0.05; "**" *p  *< 0.01; "***" for *p  *< 0.001.

In addition to qualitative and quantitative assessment of the ATF-2 activation in both cell lines, we also investigated whether ATF-2 nuclear translocation in response to SiO_2_NP was dependent on earlier upstream changes in relevant intracellular signalling mechanisms. In the model of A549 cells we investigated if SiO_2_NP exposure involved intracellular signalling cascade pathways such as JNK and/or p38. It is known that the activation of ATF-2 results from phosphorylation at Ser 69/71 by either JNK or p38 kinase [44,45]. Thus, A459 cells that had been exposed to SiO_2_NP (i.e., 80 nm) for varying time points ranging from 15 min to 24 h were analyzed for JNK or p38 activation by Western immunoblotting. We found that SiO_2_NP (80 nm) significantly increased the level of phosphorylated JNK1/2 as well as p38 in all cases when compared to untreated control cells (Figure 9A, 9B).

**Figure 10:**
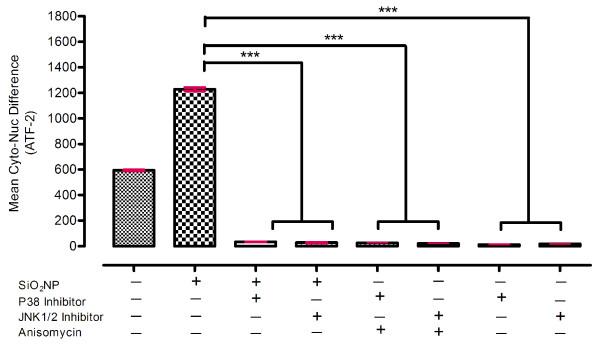
**Effect of p38 or JNK1/2 inhibition on 80 nm- size of SiO_2_NP induced ATF-2 activation**. A549 cells growing on 96-well plates were pre-treated with 10 μM specific inhibitors of p38 (SB202190), or JNK (SP600125) for 1 h and exposed to SiO_2_NP or anisomycin (positive control) for 24 h. Cells were labelled with the Cellomics^® ^HCS reagent kit for ATF-2 activation. High content screening analysis for nuclear translocation of ATF-2 was performed using an automated IN Cell Analyzer 1000 microscope equipped with IN Cell Investigator image analysis software that quantifies nuclear to cytoplasmic fluorescence intensity. Statistical analysis was carried out by Mann-whitney *U *test then used to assess the statistical significance differences. *p*-values of *p  *< 0.05; "**" *p  *< 0.01; "***" for *p  *< 0.001 were considered to be statistically significant.

We next studied whether ATF-2 nuclear translocation in response to SiO_2_NP was dependent on p38 and/or JNK activation. For this purpose, specific inhibitors of these MAPK were utilised. A pyridinyl imidazole SB202190 and an anthrapyraxolone SP600125 are well-characterised and specific inhibitors of p38 and JNK respectively [54-56]. Pre-treatment of the cells with SB202190 (10 μM) blocked SiO_2_NP-induced ATF-2 nuclear translocation (Figure 10). Similarly, pre-treatment of the cells with anthrapyraxolone SP600125 (10 μM) significantly reduced SiO_2_NP-induced ATF-2 nuclear translocation (Figure 10). Together, these results confirmed that SiO_2_NP induced ATF-2 activation was dependent on p38 and JNK, further supporting their roles in the activation of ATF-2.

## Discussion

In this study, we explored the potential cytotoxic effect of SiO_2_NP of five sizes (20, 30, 40, 80 and 400 nm), and dose-ranges from 0.01 to 0.5 mg/ml, in two cultured human cells of diverse origin: (i) a phagocytic cell line THP-1, and (ii) a lung epithelial cell line A549. We have demonstrated the cellular uptake of SiO_2_NP by confocal microscopy and HCS in both the cell lines. Active or passive transport routes of SiO_2_NP endocytosis were examined by temperature controlled assays at 37°C or 4°C, respectively. To further expand this work and gain a deeper understanding on the subtle cell stress variation at molecular level we investigated the activation of transcription factor-2 (ATF-2). To date, this is the first quantitative study showing treatment of human cell lines with SiO_2_NP (unstabilised or sodium stabilized) induces activation of ATF-2 in a dose- and time-dependent manner. This activation was found to be dependent on JNK and p38 kinase-mediated signalling pathways and we have shown that JNK and p38 are phosphorylated by SiO_2_NP treatment of cells. The involvement of p38 cell stress activated pathway is also supported by the evidence that cigarette smoke particles induced activation of the p38 MAPK inflammatory signalling and phosphorylation of its downstream ATF-2 [57].

In this work, we observed that 30 nm SiO_2_NP were more rapidly taken up by both THP-1 and A549 cells than the nominally 400 nm SiO_2_NP (made by the same synthesis route and with similar surface characteristics). Rapid internalisation was seen after 1 hr incubation at both SiO_2_NP which then turned out to be a size-dependent endocytotic process possibly associated with two competing mechanisms such as diffusion (after 1 h) and sedimentation (after 3 h). This has been previously reported by Limbach and co-workers [58] as a particle transport mechanism issue which has been associated with the nature of the cell under investigation and the quantitative treatment of the nanomaterial under investigation. A recent work from Shapero and co-workers [59], described and explained the detailed uptake and localization time course of SiO_2_NP of different sizes (50, 100 and 300 nm) by one of the cell line used, in this work A549 cells.

In agreement with previous studies [60], we have also demonstrated that the SiO_2_NP uptake was eliminated at lower temperature (4°C) in THP-1 and A549 cells [60]. Sodium azide is widely used both *in vivo *and *in vitro *as an inhibitor of cellular respiration. It acts by inhibiting cytochrome C oxidase, the last enzyme in the mitochondrial electron transport chain, and thereby produces a drop in intracellular ATP concentration [61]. The uptake of SiO_2_NP into A549 pre-treated with commonly used concentration of sodium azide (0.1%) was completely blocked thus suggesting that the uptake mechanism occurs through an energy dependent process. In contrast, this concentration of sodium azide could not efficiently inhibit SiO_2_NP uptake in THP-1 cells. Other studies have also reported on alternative uptake mechanisms such as clathrin-mediated endocytosis, caveolae/lipid raft-mediated endocytosis, macropinocytosis, or phagocytosis [62,63].

In light of this evidence, a broader understanding on the mechanism of interaction between the nanoparticles and cell lines used was also explored since each of the cell lines exhibited different responses to the various SiO_2_NP. Overall the HCS measured cytotoxicity was higher in THP-1 compared to A549 cells. In addition, THP-1 cells showed reduction in the cell viability, as shown in Figure 4. This reduction is dependent on the particle concentration, particle size, and exposure time. It has been previously reported by other researchers that the reduction of cell viability is concentration-dependent [30,64-66]. The issue of size-dependent cytotoxicity of SiO_2_NP has previously been addressed in different cell systems such as A549, Mono Mac 6 and THP-1 and EAHY926 cell lines [66,67]. It has been shown *in vivo *that amorphous SiO_2_NP are less toxic than their crystalline form [68,69].

We have also demonstrated a particle size-dependence of the cytotoxic response in THP-1 cells. As seen in Figures 4, the smaller sizes (20, and 30 nm) of the tested sodium ion stabilized, and non- stabilized SiO_2_NP at 0.5 mg/ml caused an inhibition in cell viability after 24 h incubation period. Whereas, all tested doses of the 80 and 400 nm-size SiO_2_NP caused a significant decrease in the viability of THP-1 cells. We observed that the peak of internalised 400 nm SiO_2_NP remained constant in the cytosol of THP-1 cells after 24 h exposure, while the cellular uptake level of 30 nm SiO_2_NP decreased after 24 h exposure. From this, we hypothesise that the large sized particles (400 nm SiO_2_NP) either remained within the cells or a delay in the exocytosis process induced significant decrease of the cell viability. This emphasises the need to further address the bi-directional issues (endo- and exo- cytotic process) involving the nanoparticle transport routes and mechanisms in human cells.

A549 cells are a well characterised *in vitro *model and have been extensively used for assessing cytotoxicity, including nanomaterials-induced cytotoxicity [70,71]. The findings of these works show that there was no significant reduction in cell viability caused by any of the tested SiO_2_NP at any of the doses applied (0.01, 0.1, and 0.5 mg/ml). Conversely to previous work by Lin et al. [30] which showed a significant reduction in the viability of A549 cells caused by 15 nm and 46 nm amorphous SiO_2_NP that exhibited similar cytotoxicity. Their conclusion was that the 15 nm and 46 nm SiO_2_NP aggregated to form hydrodynamic clusters of ranging sizes between 590 nm to 617 nm, respectively with subsequent toxicity due to their aggregation [30]. Conversely, we did not find a significant reduction in cell number even at the highest concentration tested (0.5 mg/ml) either by 20 nm nor 400 nm SiO_2_NP. In our study, the size and size-distribution of the SiO_2_NP used here were extensively examined and characterised across the Nanointeract consortium (Framework Programme 6 funded project), as reported by Barnes et al. [46]; this is an important consideration when using the nanoparticles for biomedical applications. In addition, our TEM and Light Scattering measurement was also carried out on retrieved 40 nm particles, and this did not show any significant aggregation.

To determine if surface coating of the SiO_2_NP affects the cellular interaction, we also evaluated the effect of alumina coating on SiO_2_NP cytotoxicity. We found that the alumina coating of positively charged SiO_2_NP did not significantly inhibit cell growth, at any dose, in any of the two cell types studied. *In vitro *studies also suggested that alumina coating pre-treatment reduced the cytotoxicity of silica [72] and mitochondrial depolarisation [73]. The mechanism by which alumina coating reduces silica toxicity is still unclear, nonetheless our latest findings are in opposition with the general perception that cationic species are more toxic to cells than anionic ones [74], and this was consistent for both the cytotoxicity and activation of ATF-2 translocation. Furthermore, it has been suggested that alumina reduces the ability of silica to generate hydroxyl radicals in the presence of hydrogen peroxide [72] and their effects are believed to alter the interaction of SiO_2_NP with cell membranes [75]. This was demonstrated in our study by the investigation of the 40 nm alumina coated SiO_2_NP which suppressed the lysosomal alterations in both THP-1 and A549 cells.

Following the uptake of SiO_2_NP, these may interact with phagolysosomal membranes leading to the release of lysosomal enzymes into the cytosol and subsequently causing cell death in phagocytic cells [76], or epithelial cells [77]. This was qualitatively and quantitatively assessed in this work. For instance, despite the reduction of cell viability of THP-1 cells at concentrations below 0.5 mg/ml, the lysosomal mass/pH changes were only noted at the highest 0.5 mg/ml of 20, 30 and 80 nm SiO_2_NP. This is in agreement with previous work by some of the authors where they demonstrated such alteration of lysosomal mass/pH in THP-1 to be linked to nanoparticles and also the cell line used [7].

**Figure 11:**
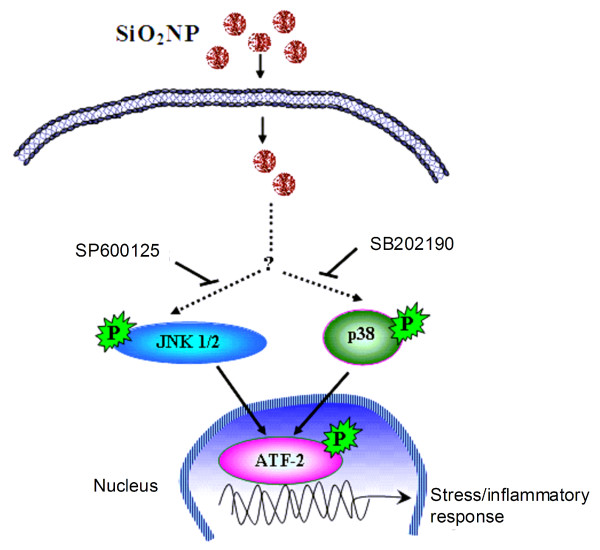
**A proposed schematic presentation of SiO_2_NP-induced stress response signalling pathway**. SiO_2_NP enter the cells by endocytosis and activate the SAPK pathway. Activated JNK and p38 further trigger the nuclear translocation of transcription factor ATF-2 with a consequent induction of stress response signalling pathways.

For the human lung epithelial cell line A549, the lysosomal mass/pH was increased following treatment with nominally 20, 80 or 400 nm particles and the latter SiO_2_NP used caused some cell biological changes (e.g. cell stress, membrane permeability, and lysosomal mass/pH), despite cell proliferation was not inhibited as shown by the cell viability measurements. In fact, it has been suggested that loss in lysosomal integrity can be attributed to apoptotic responses [78], sphingosine [79], TNF-α [80], Fas [81], lysosomal photodamage [82] or lysosomal permeability [73]. Importantly, in this study we found that alumina coated 40 nm SiO_2_NP showed lower cytotoxicity in all measured parameters and lower ATF-2 activation stress response. This might be linked to a possible reduction by the SiO_2_NP to produce hydroxyl radicals *in situ *in cells. From the results presented in this study, we propose that SiO_2_NP enter the cells by endocytosis and activate the SAPK pathway. Activated JNK and p38 further trigger the nuclear translocation of transcription factor ATF-2 with a consequent induction of stress response signalling pathways (Figure 11).

Finally, our results are in agreement with other cytotoxicity studies [31,32,83] demonstrating low cytotoxicity response induced by SiO_2_NP (below <500 nm). However, in this paper we present further evidence of gene stress response induced by these materials, which has not been previously measured by other techniques [46,61]. This opens interesting exploratory pathway where there is a pressing need for further in-depth gene-associated studies towards the elucidation of the SiO_2_NP-cells interaction mechanisms aimed at establishing the safe application of this promising nanomaterials.

## Conclusions

This study showed that at concentrations below 0.5 mg/ml of the amorphous SiO_2_NP tested (20, 30, 40, 80 and 400 nm), a low degree of cytotoxicity was observed across the two cell lines adopted (THP-1 and A549 cells). Interestingly, the cell lines did not show any significant toxic response to the alumina coated SiO_2_NP (e.g., 40 nm SiO_2_NP). In this paper, we demonstrated for the first time that SiO_2_NP are able to induce p38 and JNK MAPK activation and phosphorylation of their downstream ATF-2 target, which also clearly reflect the nature of particular cell type. In addition, we also registered a close relation between silica-induced cytotoxicity and changes to the structure or activity of detected cellular compartments (e.g., lysosomal and cell membrane) by the use of HCS which detected significant dose and particle size-dependent changes in parameters related to these compartments.

Based on our results, it may be speculated that continuous exposure to nanoparticles, even of relatively biologically "inert" nature, could impose a risk to human health. However, it may be possible to reduce such unwanted effects by surface modification of nanomaterials, for example via alumina coating of SiO_2_NP, thereby reducing the surface reactivity of the NPs. A much better mechanistic understanding of nanoparticles and their properties will support the development and evaluation of potentially reduced risk nano-products for biomedical applications, and HCS offers an ideal platform for the type of screening data needed for this purpose.

## Abbreviations

SiO_2_NP: Silica nanoparticles; HCS: high content screening; ATF-2: activation transcription factor-2; PMA: phorbol 12-myristate 13-acetate; PBS: phosphate-buffered saline; PFA: paraformaldehyde; MPCT1: multiparameter cytotoxicity assay 1.

## Competing interests

The authors declare that they have no competing interests.

## Authors' contributions

BMM aided in the conception of the study, drafted the manuscript and performed all assays. NKV, YW, AD, APM contributed to the experimental design, and made scientific contributions to the study. CE participated in the assay development. GB, BMM, APM performed the statistical analysis, and heatmaps table illustrations generations. LT, APM, and DK participated in the critical assessment of the data, and drafting of the manuscript. JH synthesised and supplied the Glantreo SiO_2_NP, and the fluorescently-labelled SiO_2_NP. AS, IL, and KD provided the silica particle characterisation, and contributed to the critical assessment of the data and drafting of the manuscript. YV supervised the study and participated in the data analysis and drafting of the manuscript. All authors have read and approved the final manuscript.

## Supplementary Material

Additional file 1 **Supplemental information to manuscript**. Light scattering measurements of alumina-coated particles (Ludox CL 420883), plot of two independent batches of Ludox particles suspended in DI water and statistical analysis tables for cell viability, membrane permeability and lysosomal mass/pH parameters. Click here for file
