# Supplementary material for: Activation of stress-related signalling pathway in human cells upon SiO2 nanoparticles exposure as an early indicator of cytotoxicity
Source: J Nanobiotechnology. 2011 Jul 29;9:29. doi: 10.1186/1477-3155-9-29 (PMC3164618; doi:10.1186/1477-3155-9-29)
Supplement: Additional file 1 — Supplemental information to manuscript. Light scattering measurements of alumina-coated particles (Ludox CL 420883), plot of two independent batches of Ludox particles suspended in DI water and statistical analysis tables for cell viability, membrane permeability and lysosomal mass/pH parameters. [file 1477-3155-9-29-S1.DOCX]

**Activation of stress-related signalling pathway in human cells upon SiO_2_ nanoparticles exposure as an early indicator of cytotoxicity**

Bashir Mustafa Mohamed^1§^, Navin Kumar Verma^1^, Adriele Prina-Mello^1,2^, Yvonne Williams^1^, Anthony M Davies^1^, Gabor Bakos^1^, Laragh Tormey^1^, Connla Edwards^1^, John Hanrahan^3^, Anna Salvati^4^, Iseult Lynch^4^, Kenneth Dawson^4^, Dermot Kelleher^1^, and Yuri Volkov^1,2^

^1^Department of clinical medicine, Institute of Molecular Medicine, Trinity College Dublin, Dublin8, Ireland

^2^Centre for Research on Adaptive Nanostructures and Nanodevices (CRANN), Naughton Institute, Trinity College Dublin, Dublin2, Ireland

^3^Glantreo Ltd., Environmental Research Institute (ERI) Building, Lee Road, Cork, Ireland

^4^Centre for BioNano Interactions, School of Chemistry and Chemical Biology, University College Dublin, Dublin4, Ireland

^§^Correspondence author: [mohamebm@tcd.ie](mailto:mohamebm@tcd.ie)

Email addresses:

BMM: [bashmohamed@gmail.com](mailto:bashmohamed@gmail.com)

NKV: [verman@tcd.ie](mailto:verman@tcd.ie)

APM: [prinamea@tcd.ie](mailto:prinamea@tcd.ie)

YW: [williamy@tcd.ie](mailto:williamy@tcd.ie)

AD: [amitche@tcd.ie](mailto:amitche@tcd.ie)

GB: [gaborgabor@gmail.com](mailto:gaborgabor@gmail.com)

LT: [tormeyla@tcd.ie](mailto:tormeyla@tcd.ie)

CE: [cedward@tcd.ie](mailto:cedward@tcd.ie)

JH: [j.hanrahan@glantreo.com](mailto:j.hanrahan@glantreo.com)

AS: [anna@fiachraucd.ie](mailto:anna@fiachraucd.ie)

IL: [iseult@fiachraucd.ie](mailto:iseult@fiachraucd.ie)

KD: [kenneth@fiachraucd.ie](mailto:kenneth@fiachra.ucd.ie)

DK: [kellehdp@tcd.ie](mailto:kellehdp@tcd.ie)

YV: [yvolkov@tcd.ie](mailto:yvolkov@tcd.ie)

**Supplementary Information**

Light Scattering particles size measurement and concentration by means of Brownian motion speed of particles in liquid suspension were carried out on alumina-coated particles (Ludox CL, 420883) by using a NanoSight LM10HS and NTA 2.1 Analytical Software (NanoSight, Wiltshire, UK). This further completes physico-chemical characterization previously reported by Barnes et al. (Nano Letters 2008, Vol 8 (9): 3069-3074, ref #[46]) on the same nanomaterials as used in this work. The hydrodynamic diameter measured by NanoSight system is in agreement with the one reported by Dynamic Light Scattering reported by Barnes et al., thus confirming the previously reported particle size and providing additional information regarding particle distribution and concentration in suspension.

**Supplemental Information figure 1.** Light scattering measurements of alumina-coated particles (Ludox CL 420883). Plot of two independent batches of Ludox particles suspended in DI water.

Table S1a: CELL VIABILITY comparative statistical analysis of SiO_2_NP (0.5 µg/ml, incubation 24h) versus their negative control. Statistical significance expressed in *p*-value.

| SiO_2_NP size [nm] or Positive control | Parameter analysed | Cell line | Time points [hours] | Percentage of variation versus negative control (%) | *p-*value  Not significant (Ns) > 0.05  * < 0.05  ** < 0.01  *** < 0.001 |
| --- | --- | --- | --- | --- | --- |
| 20 | Cell viability | THP-1 | 24 | 11.7 | 0.001 |
| 30 | Cell viability | THP-1 | 24 | 3.5 | 0.01 |
| 40 | Cell viability | THP-1 | 24 | 0.9 | Ns |
| 80 | Cell viability | THP-1 | 24 | 7.1 | 0.001 |
| 400 | Cell viability | THP-1 | 24 | 2.7 | 0.05 |
| Cysplatin | Cell viability | THP-1 | 24 | 35.4 | 0.001 |
| 20 | Cell viability | A549 | 24 | 0.5 | Ns |
| 30 | Cell viability | A549 | 24 | 1.4 | Ns |
| 40 | Cell viability | A549 | 24 | 1.0 | Ns |
| 80 | Cell viability | A549 | 24 | 0.8 | Ns |
| 400 | Cell viability | A549 | 24 | 1.4 | Ns |
| Cysplatin | Cell viability | A549 | 24 | 36.2 | 0.001 |

Table S1b: CELL VIABILITY statistical analysis of SiO_2_NP at concentration of 0.1 µg/ml, incubation 24h.

| SiO_2_NP size [nm] or Positive control | Parameter analysed | Cell line | Time points [hours] | Percentage of variation versus negative control (%) | *p-*value  Ns > 0.05  * < 0.05  ** < 0.01  *** < 0.001 |
| --- | --- | --- | --- | --- | --- |
| 20 | Cell viability | THP-1 | 24 | 7.1 | 0.001 |
| 30 | Cell viability | THP-1 | 24 | 1.5 | Ns |
| 40 | Cell viability | THP-1 | 24 | 0.9 | Ns |
| 80 | Cell viability | THP-1 | 24 | 5.8 | 0.01 |
| 400 | Cell viability | THP-1 | 24 | 4.8 | 0.01 |
| Cysplatin | Cell viability | THP-1 | 24 | 35.4 | 0.001 |
| 20 | Cell viability | A549 | 24 | 0.8 | Ns |
| 30 | Cell viability | A549 | 24 | 1.2 | Ns |
| 40 | Cell viability | A549 | 24 | 1.7 | Ns |
| 80 | Cell viability | A549 | 24 | 0.9 | Ns |
| 400 | Cell viability | A549 | 24 | 0.8 | Ns |
| Cysplatin | Cell viability | A549 | 24 | 36.2 | 0.001 |

Table S1c: CELL VIABILITY statistical analysis of SiO_2_NP at concentration of 0.01 µg/ml, incubation 24h.

| SiO_2_NP size [nm] or Positive control | Parameter analysed | Cell line | Time points [hours] | Percentage of variation versus negative control (%) | *p-*value  Ns > 0.05  * < 0.05  ** < 0.01  *** < 0.001 |
| --- | --- | --- | --- | --- | --- |
| 20 | Cell viability | THP-1 | 24 | 1.4 | Ns |
| 30 | Cell viability | THP-1 | 24 | 1.1 | Ns |
| 40 | Cell viability | THP-1 | 24 | 0.2 | Ns |
| 80 | Cell viability | THP-1 | 24 | 3.2 | 0.05 |
| 400 | Cell viability | THP-1 | 24 | 4.4 | 0.01 |
| Cysplatin | Cell viability | THP-1 | 24 | 35.4 | 0.001 |
| 20 | Cell viability | A549 | 24 | 0.9 | Ns |
| 30 | Cell viability | A549 | 24 | 0.9 | Ns |
| 40 | Cell viability | A549 | 24 | 1.3 | Ns |
| 80 | Cell viability | A549 | 24 | 0.3 | Ns |
| 400 | Cell viability | A549 | 24 | 0.4 | Ns |
| Cysplatin | Cell viability | A549 | 24 | 36.2 | 0.001 |

Table S2a: MEMBRANE PERMEABILITY comparative statistical analysis of SiO2NP (0.5 µg/ml, incubation 24h) versus their negative control. Statistical significance expressed in *p-*value.

| SiO_2_NP size [nm] or Positive control | Parameter analysed | Cell line | Time points [hours] | Percentage of variation versus negative control (%) | *p-*value  Ns > 0.05  * < 0.05  ** < 0.01  *** < 0.001 |
| --- | --- | --- | --- | --- | --- |
| 20 | Membrane Permeability | THP-1 | 24 | 4.3 | 0.01 |
| 30 | Membrane Permeability | THP-1 | 24 | 4.1 | 0.01 |
| 40 | Membrane Permeability | THP-1 | 24 | 0.8 | Ns |
| 80 | Membrane Permeability | THP-1 | 24 | 3.0 | 0.05 |
| 400 | Membrane Permeability | THP-1 | 24 | 0.3 | Ns |
| Cysplatin | Membrane Permeability | THP-1 | 24 | 35.4 | 0.001 |
| 20 | Membrane Permeability | A549 | 24 | 1.1 | Ns |
| 30 | Membrane Permeability | A549 | 24 | 2.4 | Ns |
| 40 | Membrane Permeability | A549 | 24 | 0.4 | Ns |
| 80 | Membrane Permeability | A549 | 24 | 2.3 | Ns |
| 400 | Membrane Permeability | A549 | 24 | 3.3 | 0.05 |
| Cysplatin | Membrane Permeability | A549 | 24 | 36.2 | 0.001 |

Table S2b: MEMBRANE PERMEABILITY statistical analysis of SiO_2_NP at concentration of 0.1 µg/ml, incubation 24h.

| SiO_2_NP size [nm] or Positive control | Parameter analysed | Cell line | Time points [hours] | Percentage of variation versus control (%) | *p-*value  Ns > 0.05  * < 0.05  ** < 0.01  *** < 0.001 |
| --- | --- | --- | --- | --- | --- |
| 20 | Membrane Permeability | THP-1 | 24 | 2.7 | Ns |
| 30 | Membrane Permeability | THP-1 | 24 | 1.3 | Ns |
| 40 | Membrane Permeability | THP-1 | 24 | 0.5 | Ns |
| 80 | Membrane Permeability | THP-1 | 24 | 1.3 | Ns |
| 400 | Membrane Permeability | THP-1 | 24 | 0.7 | Ns |
| Cysplatin | Membrane Permeability | THP-1 | 24 | 35.4 | 0.001 |
| 20 | Membrane Permeability | A549 | 24 | 1.2 | Ns |
| 30 | Membrane Permeability | A549 | 24 | 1.6 | Ns |
| 40 | Membrane Permeability | A549 | 24 | 0.3 | Ns |
| 80 | Membrane Permeability | A549 | 24 | 2.0 | Ns |
| 400 | Membrane Permeability | A549 | 24 | 0.7 | Ns |
| Cysplatin | Membrane Permeability | A549 | 24 | 36.2 | 0.001 |

Table S2c: MEMBRANE PERMEABILITY statistical analysis of SiO_2_NP at concentration of 0.01 µg/ml, incubation 24h.

| SiO_2_NP size [nm] or Positive control | Parameter analysed | Cell line | Time points [hours] | Percentage of variation versus negative control (%) | *p-*value  Ns > 0.05  * < 0.05  ** < 0.01  *** < 0.001 |
| --- | --- | --- | --- | --- | --- |
| 20 | Membrane Permeability | THP-1 | 24 | 0.3 | Ns |
| 30 | Membrane Permeability | THP-1 | 24 | 0.6 | Ns |
| 40 | Membrane Permeability | THP-1 | 24 | 0.4 | Ns |
| 80 | Membrane Permeability | THP-1 | 24 | 0.2 | Ns |
| 400 | Membrane Permeability | THP-1 | 24 | 0.4 | Ns |
| Cysplatin | Membrane Permeability | THP-1 | 24 | 35.4 | 0.001 |
| 20 | Membrane Permeability | A549 | 24 | 1.3 | Ns |
| 30 | Membrane Permeability | A549 | 24 | 1.4 | Ns |
| 40 | Membrane Permeability | A549 | 24 | 0.6 | Ns |
| 80 | Membrane Permeability | A549 | 24 | 1.8 | Ns |
| 400 | Membrane Permeability | A549 | 24 | 0.5 | Ns |
| Cysplatin | Membrane Permeability | A549 | 24 | 36.2 | 0.001 |

Table S3a: LYSOSOMAL MASS comparative statistical analysis of SiO2NP (0.5 µg/ml, incubation 24h) versus their negative control. Statistical significance expressed in *p*-value.

| SiO_2_NP size [nm] or Positive control | Parameter analysed | Cell line | Time points [hours] | Percentage of variation versus negative control (%) | *p-*value  Ns >0.05  * > 0.05  ** > 0.01  *** >0.001 |
| --- | --- | --- | --- | --- | --- |
| 20 | Lysosomal mass | THP-1 | 24 | 5.2 | 0.01 |
| 30 | Lysosomal mass | THP-1 | 24 | 5.2 | 0.01 |
| 40 | Lysosomal mass | THP-1 | 24 | 0.8 | Ns |
| 80 | Lysosomal mass | THP-1 | 24 | 7.1 | 0.001 |
| 400 | Lysosomal mass | THP-1 | 24 | 0.8 | Ns |
| Cysplatin | Lysosomal mass | THP-1 | 24 | 35.4 | 0.001 |
| 20 | Lysosomal mass | A549 | 24 | 4.2 | 0.01 |
| 30 | Lysosomal mass | A549 | 24 | 1.0 | Ns |
| 40 | Lysosomal mass | A549 | 24 | 0.6 | Ns |
| 80 | Lysosomal mass | A549 | 24 | 4.5 | 0.01 |
| 400 | Lysosomal mass | A549 | 24 | 5.7 | 0.001 |
| Cysplatin | Lysosomal mass | A549 | 24 | 36.2 | 0.001 |

Table S3b: LYSOSOMAL MASS statistical analysis of SiO_2_NP at concentration of 0.1 µg/ml, incubation 24h.

| SiO_2_NP size [nm] or Positive control | Parameter analysed | Cell line | Time points [hours] | Percentage of variation versus negative control (%) | *p-*value  Ns >0.05  * > 0.05  ** > 0.01  *** >0.001 |
| --- | --- | --- | --- | --- | --- |
| 20 | Lysosomal mass | THP-1 | 24 | 1.7 | Ns |
| 30 | Lysosomal mass | THP-1 | 24 | 5.4 | 0.01 |
| 40 | Lysosomal mass | THP-1 | 24 | 2.6 | Ns |
| 80 | Lysosomal mass | THP-1 | 24 | 7.4 | 0.001 |
| 400 | Lysosomal mass | THP-1 | 24 | 1.0 | Ns |
| Cysplatin | Lysosomal mass | THP-1 | 24 | 35.4 | 0.001 |
| 20 | Lysosomal mass | A549 | 24 | 0.2 | Ns |
| 30 | Lysosomal mass | A549 | 24 | 1.1 | Ns |
| 40 | Lysosomal mass | A549 | 24 | 0.7 | Ns |
| 80 | Lysosomal mass | A549 | 24 | 1.3 | Ns |
| 400 | Lysosomal mass | A549 | 24 | 3.2 | 0.01 |
| Cysplatin | Lysosomal mass | A549 | 24 | 36.2 | 0.001 |

Table S3c: LYSOSOMAL MASS statistical analysis of SiO_2_NP at concentration of 0.01 µg/ml, incubation 24h.

| SiO_2_NP size [nm] or Positive control | Parameter analysed | Cell line | Time points [hours] | Percentage of variation versus negative control (%) | *p-*value  Ns >0.05  * > 0.05  ** > 0.01  *** >0.001 |
| --- | --- | --- | --- | --- | --- |
| 20 | Lysosomal mass | THP-1 | 24 | 0.3 | Ns |
| 30 | Lysosomal mass | THP-1 | 24 | 2.2 | Ns |
| 40 | Lysosomal mass | THP-1 | 24 | 2.5 | Ns |
| 80 | Lysosomal mass | THP-1 | 24 | 4.2 | 0.01 |
| 400 | Lysosomal mass | THP-1 | 24 | 1.0 | Ns |
| Cysplatin | Lysosomal mass | THP-1 | 24 | 35.4 | 0.001 |
| 20 | Lysosomal mass | A549 | 24 | 0.3 | Ns |
| 30 | Lysosomal mass | A549 | 24 | 0.2 | Ns |
| 40 | Lysosomal mass | A549 | 24 | 0.2 | Ns |
| 80 | Lysosomal mass | A549 | 24 | 0.2 | Ns |
| 400 | Lysosomal mass | A549 | 24 | 2.4 | Ns |
| Cysplatin | Lysosomal mass | A549 | 24 | 36.2 | 0.001 |
